# The Impact of EAT, NYHA Functional Class, and Inflammation on VT/VF in NICMP Patients

**DOI:** 10.1016/j.jacadv.2025.101981

**Published:** 2025-07-11

**Authors:** Mehmet M. Tabakci, Yusuf Aslantas

**Affiliations:** a Independent Researcher, Rastatt, Germany; b Gemeinschaftskrankenhaus Havelhöhe Klinik für Anthroposopische Medizin, Berlin, Germany

We read with great interest the recent paper by Nakomori et al.^1^ In this letter, we aim to highlight certain methodological and clinical aspects of the study, offering a different perspective and contributing to the ongoing discourse. In patients with nonischemic cardiomyopathy, NYHA functional class and ejection fraction are well-established prognostic indicators, strongly associated with life-threatening arrhythmias such as ventricular tachycardia and ventricular fibrillation (VT/VF). In our own study, we demonstrated for the first time the relationship between a high NYHA functional class and low epicardial adipose tissue (EAT) thickness in this patient group.^2^ Based on this evidence, we suggest that incorporating patients with NYHA functional class IV, which were not included in the aforementioned study, and performing a subgroup analysis according to NYHA classification could unveil a stronger relationship between EAT and arrhythmias.

Secondly, the absence of ejection fraction as an independent predictor for VT/VF in this study could be explained by the inclusion of patients with hypertrophic cardiomyopathy and sarcoidosis, where inflammation and fibrosis are dominant. Moreover, the stronger association between VT/VF and the sarcoidosis group suggests that these arrhythmias may be primarily driven by inflammation. In light of this, excluding these subgroups or conducting subgroup analysis may provide a clearer and more significant demonstration of the pure EAT-VT/VF relationship in nonischemic cardiomyopathy patients.

Finally, in sarcoidosis patients—an inflammatory disease known to be closely linked with severe arrhythmias—the independent association between VT/VF and EAT thickness could suggest that assessing the content of EAT rather than its thickness may be more insightful. In this context, the inclusion of inflammatory and arrhythmogenic markers such as adrenomedullin and adiponectin, derived from EAT, alongside EAT thickness, could strengthen the study by incorporating indexed parameters for risk stratification. This approach would provide a more comprehensive view and potentially enhance the clinical value of the study.
